# lncRNA HOTAIR promotes ROS generation and NLRP3 inflammasome activation by inhibiting Nrf2 in diabetic retinopathy

**DOI:** 10.1097/MD.0000000000035155

**Published:** 2023-09-15

**Authors:** Hui You, Hongyu Li, Wenjun Gou

**Affiliations:** a Department of Ophthalmology, Suining Central Hospital, Suining, China; b Department of gynaecology, Suining Central Hospital, Suining, China.

**Keywords:** diabetic retinopathy, HOTAIR, lncRNA, NLRP3 inflammasome, Nrf2, ROS

## Abstract

**Background:**

Diabetic retinopathy (DR) is a microvascular complication associated with damage to the retina due to inflammation induced by high glucose. Activation of the NLRP3 inflammasome plays a critical role in DR and its prevention is beneficial to patients. However, the regulation of long non-coding RNA (lncRNA) in NLRP3 inflammasome activation of DR is incompletely understood. So, this study aimed to uncover the functional and regulatory mechanism of the lncRNA HOTAIR in NLRP3 inflammasome activation in Dr

**Methods:**

The vitreous humor was collected from the patients and detected the inflammatory and oxidative stress makers. Human retinal endothelial cells (HRECs) were cultured and stimulated in low D-glucose (5 mmol/L) or high D-glucose (20 mmol/L). Additionally, HRECs were knocked down HOTAIR with a si-RNA. Then, the NLRP3 inflammasome activation was analyzed by western blotting and pyroptosis cell imaging. The ROS was measured by specific probe. The activation of Nrf2 measured by Immunofluorescent staining. The interaction between HOTAIR and Nrf2 was evaluated by co-immunoprecipitation and RNA immunoprecipitation.

**Results:**

The expression of HOTAIR was significantly increased in the vitreous of patients with DR and in HRECs stimulated with high glucose. Furthermore, HOTAIR knockdown relieved NLRP3 inflammasome activation. More specifically, HOTAIR knockdown suppressed the expression of NLRP3, pro-caspase-1, and pro-IL-1β, as well as IL-1β maturation and pyroptosis. HOTAIR knockdown also interfered with the ROS generation induced by high glucose. Moreover, HOTAIR promoted the interaction between Nrf2 and Keap1 by binding and inactivating Nrf2.

**Conclusion:**

The lncRNA HOTAIR promotes NLRP3 inflammasome activation and ROS generation by inhibiting Nrf2 in Dr

## 1. Introduction

Diabetic retinopathy (DR), a health problem caused by the high morbidity associated with diabetes, has affected millions worldwide.^[1]^ Hyperglycemia results in the alteration of biochemical and metabolic pathways and inflammatory responses, thus promoting vascular damage and chronic complications, including Dr^[2,3]^ Attenuating the inflammation induced by hyperglycemia in the retina may be an effective treatment for DR.^[4]^ Therefore, clarifying the mechanism of the inflammatory response in DR may help develop effective therapeutic targets and bring benefits to patients.

The NLR family pyrin domain-containing 3 (NLRP3) inflammasome plays an important role in carcinogenesis and the progression of inflammation. Inappropriate activation of the NLRP3 inflammasome has been shown in a variety of diseases, including Dr^[5–7]^ Upon activation, the activated NLRP3 inflammasome serves as a platform for the production of inflammatory factors, a phenomenon that precedes the characteristic pathological changes seen in Dr These inflammatory factors mediate the dysfunction and proliferation of retinal endothelial cells and contribute to abnormal neovascularization.^[8]^

Oxidative stress plays a pivotal role in diabetes and its complications.^[9]^ Reactive oxygen species (ROS), which are derived from oxidative stress, are critical signaling intermediates that drive NLRP3 inflammasome activation and ROS participate in the inflammatory response in Dr^[9,10]^ Hyperglycemia induces mitochondrial damage and NADPH oxidase activation, which enhances ROS generation and can exacerbate the inflammatory microenvironment of retinal endothelial cells.^[10,11]^ Oxidative stress is also induced by the damage of intracellular antioxidant defense system, which neutralizes ROS. The promoters of the genes that encode antioxidants including glutathione peroxidase (GPx), heme oxygenase-1 (HO-1), and superoxide dismutase (SOD), contain an antioxidant element, which is in turn controlled by nuclear factor E2-related factor 2 (Nrf2).^[12,13]^ Nrf2 is a classical regulator of antioxidants and the inactivation of Nrf2 has been detected in various models of oxidative stress.^[14,15]^

With the development of gene technology, long non-coding RNAs (lncRNAs) have been identified, which are larger than 200 base pairs and have little protein-coding capacity. However, lncRNAs influence the transcription of target genes by direct interaction with the promoter of the target DNA or by regulating the production of transcription factors.^[16,17]^ In addition, lncRNAs facilitate chromatin remodeling.^[16]^ They can affect the expression of genes involved in pathophysiological processes of diseases, including Dr^[18,19]^ The lncRNA HOX transcript antisense intergenic RNA (HOTAIR) is a conserved lncRNA that participates in many pathological processes, including tumors,^[20]^ inflammation,^[21]^ and diabetes.^[22]^ A recent study reported that HOTAIR is involved in oxidative stress and angiogenesis in retinal endothelial cells.^[23]^ However, the role of HOTAIR in the modulation of the NLRP3 inflammasome in DR remains unclear.

The goal of our study was to explore the role of the lncRNA HOTAIR in the development of Dr We evaluated whether HOTAIR promoted the NLRP3 inflammasome activation induced by high glucose, specifically via the regulation of oxidative stress by inhibiting Nrf2.

## 2. Materials and Methods

### 2.1. Clinical samples

A total of 22 patients with DR undergoing a pars plana vitrectomy at Suining Central Hospital from January 2021 to June 2022 were enrolled in this study, 39 to 68 years old. The inclusion criteria were individuals diagnosed with DR and type 2 diabetes mellitus, and adult. The exclusion criteria were retinopathy caused by hypertension or trauma, and retinal detachment. As controls, age- and sex-matched diabetic patients (n = 10) and non-diabetic patients (n = 10) undergoing a pars plana vitrectomy during this period also were enrolled in our study, 41 to 66 years old. Vitreous humor tissue was collected from the patients. This study was approved by the Ethics Committee of Suining Central Hospital (NO: LLSLH20220087), and all patients provided written informed consent.

### 2.2. Cell culture

Human retinal endothelial cells (HRECs) (Shanghai Fumeng Gene Biotechnology Co., Ltd, Shanghai, China, cell number: 6530) were grown in DMEM culture medium supplemented with 10% FBS and 1% penicillin/streptomycin (PYG0016; Boster Bio, Wuhan, China). Cells were cultured in a humidified atmosphere containing 5% CO_2_/95% air at 37°C. HRECs were grown to 80% and 90% confluence then serum-starved for 24 h before exposure to glucose or siRNA treatments. HRECs from the fifth to eighth passage were stimulated in low D-glucose (5 mmol/L) or high D-glucose (20 mmol/L) for 96 hours, as described previously.^[24]^

### 2.3. Measurement of cytokine and redox markers

Cytokine levels in the vitreous humor or conditioned media were detected using ELISA kits following the manufacturer’s instructions, including IL-1β (EMC001b; NeoBioScience, Shenzhen, China) and IL-18 (EHC127.48; NeoBioScience). Vitreous humor malondialdehyde (MDA) activity was measured using the thiobarbituric acid method (S0131S; Beyotime Biotechnology, Shanghai, China), and SOD activity was measured using the xanthine oxidase method (S0101S; Beyotime Biotechnology).

### 2.4. Gene silencing and treatment

HRECs were seeded in 6-well plates or on slides and then transfected with lncRNA HOTAIR siRNA or negative control siRNA (GenePharma, Shanghai, China) using Lipofectamine RNAiMAX transfection reagent (13778-030; Invitrogen, Carlsbad, CA) according to the manufacturers’ instructions. In addition, HRECs were transfected with siRNA Nrf2 or overexpressing-Nrf2 (oe-HOTAIR) using Lipofectamine 2000 (12566014; Invitrogen) according to the manufacturer’s instructions. The sequences of siRNA are shown in Table 1.

**Table 1:** List of small interfering RNA (siRNA) sequences used in the present study.

	Sense (5′-3′)	Antisense (5′-3′)
si-HOTAIR	CCCAUGGACUCAUAAACAATT	UUGUUUAUGAGUCCAUGGGTT
si-Nrf2	GAGUAAGUCGAGAAGUAUU	AAUACUUCUCGACUUACUC
si-NC	UUCUCCGAACGUGUCACGUTT	ACGUGACACGUUCGGAGAATT

### 2.5. Quantitative Real-Time PCR

As described previously,^[24,25]^ the total RNA was extracted from samples with TRIzol reagent (15596026; Thermo Fisher, Waltham, MA). Total RNA was reverse transcribed into complementary DNA (cDNA) with a reverse transcriptase kit (2690S; Takara, Tokyo, Japan). qRT-PCR was performed using a real-time system with SYBR Green Master Mix (3735S; Takara). The β-actin gene was used as an endogenous control. The relative gene expression levels were analyzed by the 2^−ΔΔCT^ method. The primer sequences (Sangon Biotech, Shanghai, China) are shown in Table 2.

**Table 2:** The sequences of this study.

Gene	Sequence	
LncRNA HOTAIR	Forward sequence (5′–3′)	CAGTGGGGAACTCTGACTCG
	Reverse sequence (5′–3′)	GTGCCTGGTGCTCTCTTACC
NLRP3	Forward sequence (5′–3′)	GATCTTCGCTGCGATCAACAG
	Reverse sequence (5′–3′)	CGTGCATTATCTGAACCCCAC
Pro-caspase-1	Forward sequence (5′–3′)	AGAGGATTTCTTAACGGATGCA
	Reverse sequence (5′–3′)	TCACAAGACCAGGCATATTCTT
Pro-IL-1β	Forward sequence (5′–3′)	TCGCAGCAGCACATCAACAAGAG
	Reverse sequence (5′–3′)	TGATCATGTCCTCATCCTGGAAGG
HO-1	Forward sequence (5′–3′)	CCCACCAAGTTCAAACAGCTC
	Reverse sequence (5′–3′)	AGGAAGGCGGTCTTAGCCTC
SOD	Forward sequence (5′–3′)	CACTGCAAGGAACAACAGGC
	Reverse sequence (5′–3′)	ACCAGGCTTGATGCACATCTT
NQO1	Forward sequence (5′–3′)	CGCAGACCTTGTGATA
	Reverse sequence (5′–3′)	TGGCAGCGTAAGTGTA
GPx	Forward sequence (5′–3′)	TTATTAACGATGTCCAACCCGTC
	Reverse sequence (5′–3′)	CCAGAGCTATGCCAACAAAATCT
Nrf2	Forward sequence (5′–3′)	TGCTTTATAGCGTGCAAACCTCGC
	Reverse sequence (5′–3′)	AATCCAT GTCCCT TGACAGCACAGA
Keap1	Forward sequence (5′–3′)	GTGTCCATTGAGGGTATCCACC
	Reverse sequence (5′–3′)	GCTCAGCGAAGTTGGCGAT
β-actin	Forward sequence (5′–3′)	AGCCTCGCCTTTGCCGA
	Reverse sequence (5′–3′)	CTGGTGCCTGGGGCG

### 2.6. Western blotting

Proteins from cells or supernatants were prepared as previously described^[26,27]^ or using a nuclear and cytoplasmic protein extraction kit (P0028; Beyotime Biotechnology) according to the manufacturer’s instructions. The detection of protein form supernatants is same in all groups to ensure the loading protein level is coincident. Proteins were separated, transferred, and blocked, after which membranes were incubated with the following antibodies: anti-IL-1β (1:1000; WL02257, WanleiBio, Shenyang, China), anti-Caspase-1 (1:1000; WL03325, WanleiBio), anti-NLRP3 (1:1000; WL02635, WanleiBio), anti-GSDMD (1:1000; 39754; Cell Signaling Technology, Danvers, MA), anti-GAPDH (1:5000; GTX100118, GeneTex, CA), anti-Nrf2 (1:1000; sc-365949, Santa, Dallas, TX), anti-HO-1 (1:1000; WL02400, WanleiBio), anti-Keap1 (1:1000; WL03285, WanleiBio), anti-NQO1 (1:1000; YT3186, Immunoway, Suzhou, China), anti-SOD2 (1:1000; WL02506, WanleiBio), and anti-GPX (1:1000; WL02497a, WanleiBio).

### 2.7. Pyroptosis cell imaging

HRECs were seeded and treated in 24-well plates. HRECs were stained with 8 μg/mL propidium iodide (PI) and 8 μg/mL Hoechst dye (C0003; Beyotime Biotechnology), and then imaged using a fluorescent microscope (Olympus, Tokyo, Japan). PI-positive cells indicated cell death.

### 2.8. Measurement of cellular ROS

HRECs were seeded and treated in 12-well plates. Cells were incubated with 10 μM 2,7-dichlorodihydrofluorescein diacetate (DCFH-DA; S0033S; Beyotime Biotechnology) for 30 minutes at 37°C. Images of cell loaded probes were captured using a fluorescent microscope and fluorescence intensity was measured using a microplate reader.

### 2.9. Detection of mitochondrial ROS

Mitochondrial ROS of HRECs were assessed using a MitoSOX kit (M36005; Invitrogen). Mito-Track Green (C1048; Beyotime Biotechnology) staining was used for mitochondrial localization. The fluorescence intensity was analyzed using confocal laser scanning microscopy.

### 2.10. Immunofluorescent staining

HRECs were fixed with 4% ice-cold paraformaldehyde for 20 minutes and blocked with 1% BSA containing 0.3% Triton X-100 for 1 hour, after which the cells were incubated with anti-Nrf2 antibody (1:100) overnight at 4°C, and subsequently stained with Alexa Fluor 488 goat-anti-mouse IgG for 1 hour and stained with DAPI. The fluorescence was analyzed using confocal laser scanning microscopy.

### 2.11. Co-immunoprecipitation

HRECs were lysed and the lysate was collected and incubated with anti-Keap1 antibody (1:100) overnight at 4°C to obtain antibody-target protein complexes and the complexes were gathered with Protein G agarose beads for 2 hours at 4°C. The sediment was resuspended in 1 × SDS loading buffer and analyzed by western blotting.

### 2.12. RNA immunoprecipitation

The interaction between HOTAIR and Nrf2 was evaluated using the ChIP-IT (P2078; Beyotime Biotechnology) and qRT-PCR. Protein-RNA complexes were immunoprecipitated by an antibody specific to Nrf2 and normal rabbit IgG was used as a control. The complex was decrosslinked and extracted, and then cDNA was synthesized using a high-capacity cDNA reverse transcription kit (cat. no. 4368814; Applied Biosystems).^[24]^ The lncRNA HOTAIR level was quantified by qRT-PCR.

### 2.13. Statistical analysis

The data is presented as the mean ± SEM or the median with an interquartile range. Statistical analysis was performed using one-way analysis of variance (ANOVA) followed by least significant difference (LSD) analysis, the signed-rank test, or Spearman correlation analysis. A *P* value of < .05 was considered to be statistically significant.

## 3. Results

### 3.1. The lncRNA HOTAIR is upregulated in the vitreous of patients with diabetic retinopathy

**Figure 1.**
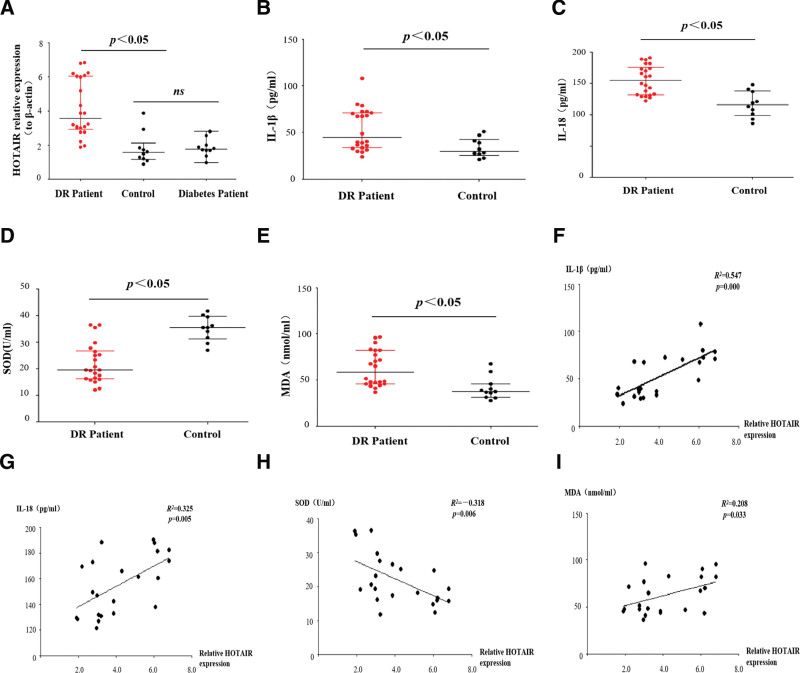
The lncRNA HOTAIR is upregulated in the vitreous of patients with diabetic retinopathy. (A) The levels of HOTAIR in the vitreous humor of patients with DR (n = 22), controls (n = 10), and diabetes patients (n = 10). (B–E) The levels of IL-1β, IL-18, MDA, and SOD in the vitreous humor of patients with DR and controls. (F–I) Correlation analysis between HOTAIR and IL-1β, IL-18, MDA, and SOD in patients with DR Data are presented as the median with the interquartile range. DR = diabetic retinopathy, lncRNA = long non-coding RNA, MDA = malondialdehyde, SOD = superoxide dismutase.

To explore the role of the lncRNA HOTAIR in DR, we determined the levels of HOTAIR, inflammatory cytokines, and markers of oxidative stress in the vitreous humor of patients with DR. HOTAIR expression was significantly increased in the vitreous humor of patients with DR compared to controls (Fig. 1A). In addition, the levels of IL-1β, IL-18, and MDA were upregulated, while the level of SOD was downregulated in the vitreous humor of patients with DR (Fig. 1B–E). There was a positive correlation between HOTAIR and IL-1β, IL-18, and MDA, and a negative correlation between HOTAIR and SOD (Fig. 1F–I). These data suggest that the lncRNA HOTAIR may contribute to DR by increasing oxidative stress and inflammation.

### 3.2. High glucose increases HOTAIR in human retinal endothelial cells

**Figure 2.**
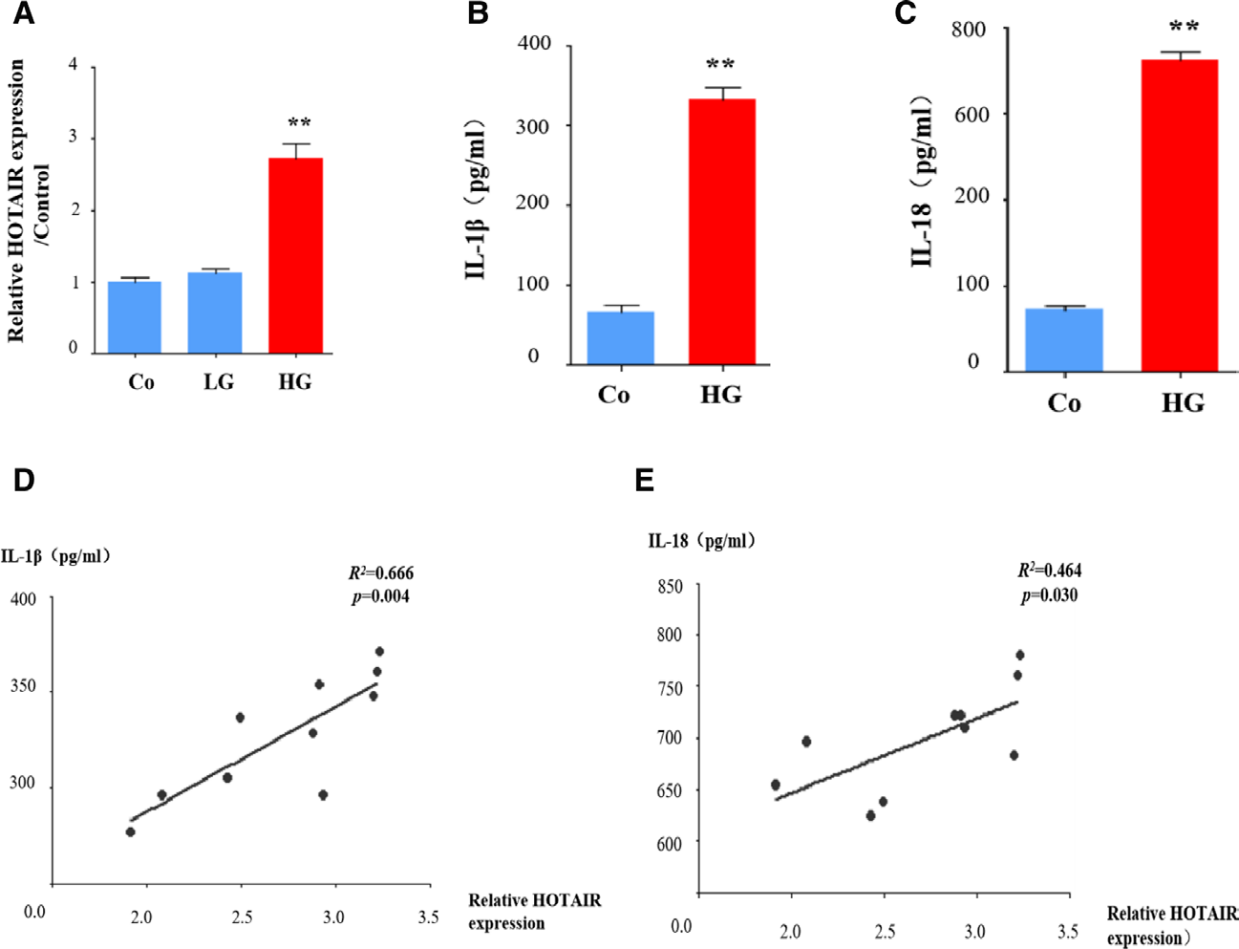
High glucose increases HOTAIR in HRECs. HRECs were incubated with high glucose (HG), low glucose (LW), or normal glucose (Co). (A) HOTAIR levels were analyzed by qRT-PCR. (B and C) Supernatants were analyzed by ELISA to measure IL-1β and IL-18 levels. (D and E) Correlation analysis between HOTAIR and IL-1β and IL-18. Data are presented as the mean ± SEM, ^**^P < .01 compared with the control group. HRECs = human retinal endothelial cells.

Next, we determined the levels of HOTAIR in vitro. Incubation with high glucose increased the levels of HOTAIR in HRECs compared with controls (Fig. 2A). However, low glucose did not markedly enhance the levels of HOTAIR (Fig. 2A). As HOTAIR appears to be closely related to inflammatory cytokines in patients with DR (Fig. 1), we investigated inflammatory markers in a cell model. High glucose led to significant increases in IL-1β and IL-18 in HRECs (Fig. 2B and C). Furthermore, there were positive correlations between HOTAIR and IL-1β and IL-18 (Fig. 2D and E). These results are in accordance with those shown in Figure 1.

### 3.3. HOTAIR promotes NLRP3 inflammasome activation induced by high glucose in HRECs

**Figure 3.**
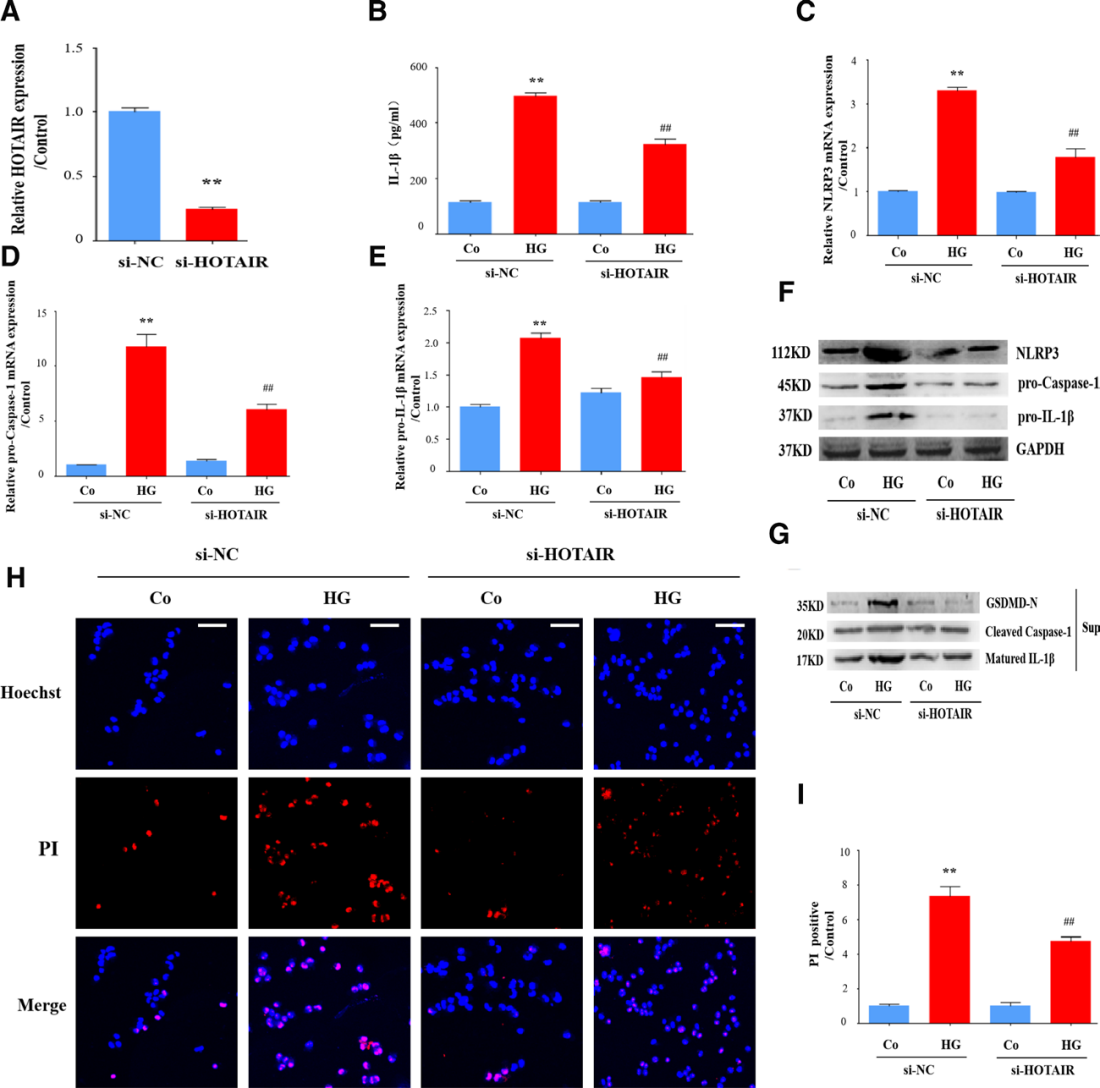
HOTAIR promotes activation of the NLRP3 inflammasome induced by high glucose in HRECs. HRECs were transfected with siRNA-HOTAIR or siRNA-NC (A) and then incubated with high glucose (HG). (B) Supernatants were analyzed by ELISA to measure IL-1β (n = 3). (C–E). The NLRP3, pro-caspase-1, and pro-IL-1β mRNAs were detected by qRT-PCR (n = 3). (F) Cell lysates were analyzed by immunoblotting to determine the NLRP3, pro-caspase-1, and pro-IL-1β protein expression. (G) Supernatants were analyzed by immunoblotting to determine the release of cleaved caspase-1 and mature IL-1β, and GSDMD-N. (H–I) Cell death was detected by propidium iodide (PI) and Hoechst 33342 staining (n = 3). Scale bars, 100 µm. Data are presented as the mean ± SEM, ^**^P < .01 compared with the control group, ^##^*P* < .01 compared with the si-NC group. HRECs = human retinal endothelial cells, Nrf2 = nuclear factor E2-related factor 2.

Inflammatory cytokines mediated by the NLRP3 inflammasome participate in the progression of DR.^[7,28,29]^ Activation of the NLRP3 inflammasome promotes the maturation and secretion of IL-1β in DR. ^[30,31]^ Therefore, we examined the role of HOTAIR in regulating the NLRP3 inflammasome by a strategy that knock down HOTAIR with a si-RNA (Fig. 3A). As shown in Figure 3B, HOTAIR knockdown depressed the IL-1β levels induced by high glucose, suggesting that HOTAIR may regulate NLRP3 inflammasome activation.

Activation of the NLRP3 inflammasome is a complicated procedure involving the upregulation of NLRP3, pro-caspase-1, and pro-IL-1β, leading to the recruitment of cleaved caspase-1 and the release of mature IL-1β.^[14]^ First, we determined the inflammasome expression and found that high glucose elevated expression of NLRP3, pro-caspase-1, and pro-IL-1β at both the mRNA and protein level; however, knockdown of HOTAIR ameliorated this increase (Fig. 3C–F). Cleaved caspase-1 and mature IL-1β may be released into the culture medium and these levels indicate the extent of NLRP3 inflammasome activation.^[30]^ Thus, we determined the level of cleaved caspase-1 and mature IL-1β in cell supernatants by western immunoblotting. High glucose promoted an increase in the release of cleaved caspase-1 and mature IL-1β but this increase was suppressed by HOTAIR knockdown (Fig. 3G). In addition to promoting IL-1β release, activation of the NLRP3 inflammasome also causes pyroptosis, an inflammatory death of cells.^[32]^ We used propidium iodide uptake (red fluorescence) to directly evaluate the inflammatory death of HRECs. As shown in Figure 3H and I, knocking down HOTAIR relieved the cell death induced by high glucose. GSDMD is the execution protein for pyroptosis and the activated N-terminal fragment (GSDMD-N) is responsible for pyroptosis.^[32]^ HOTAIR knockdown inhibited the increase in GSDMD-N induced by high glucose (Fig. 3G). These findings indicate that the lncRNA HOTAIR is at least partially responsible for the activation of the NLRP3 inflammasome in DR.

### 3.4. HOTAIR mediates oxidative stress triggered by high glucose in HRECs

**Figure 4.**
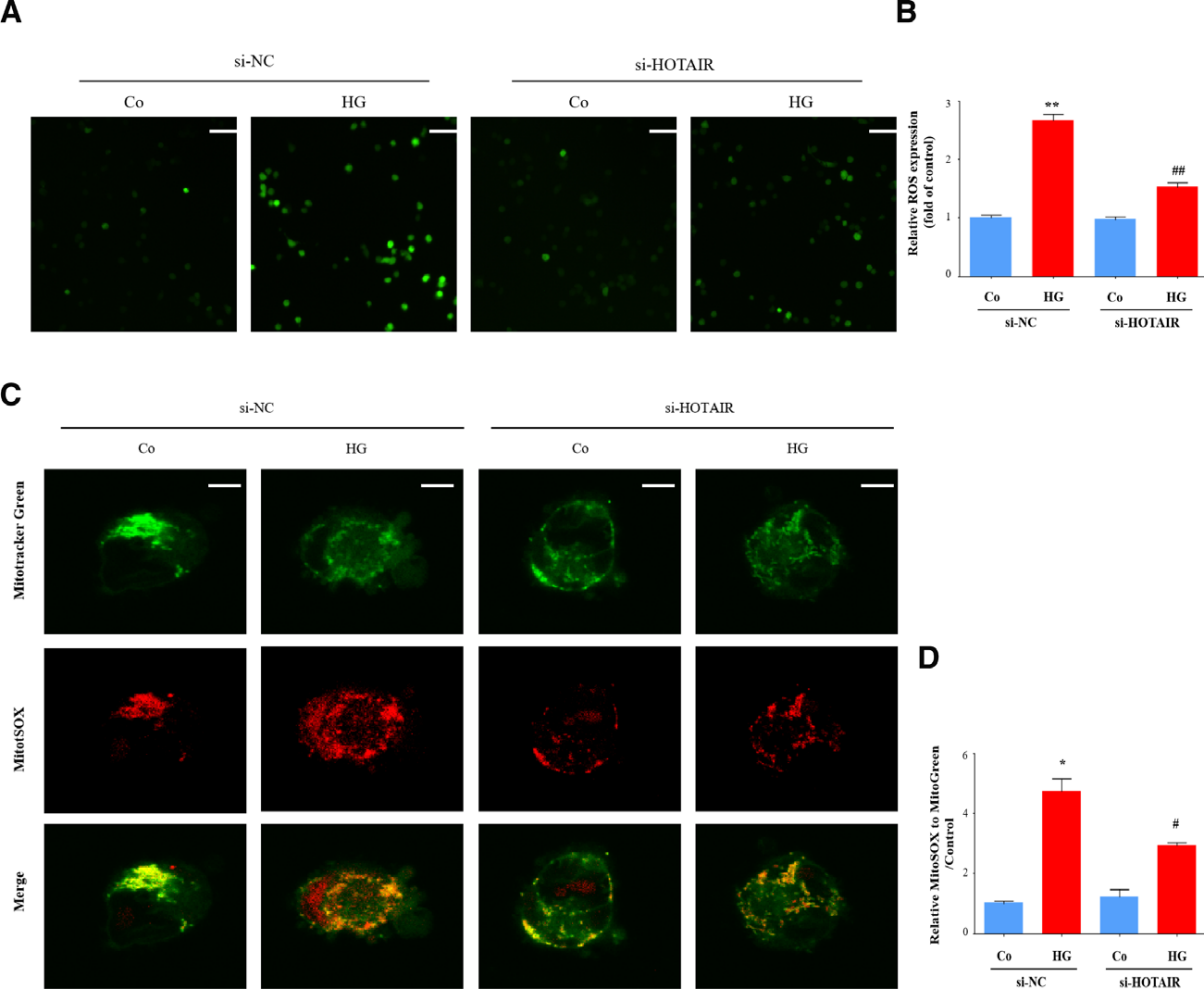
HOTAIR mediates oxidative stress. HRECs were transfected with siRNA-HOTAIR or siRNA-NC, and then incubated with high glucose (HG). (A and B) Cells were stained with DCFH (10 μM) and then analyzed by florescent microscopy and using a microplate reader (n = 3). Scale bars, 50 µm. (C and D) Cells were stained with MitoSOX (Red) and MitoTracker (Green) to determine mitochondrial ROS levels. Scale bars, 5 µm. The relative fluorescence intensity of MitoSOX to MitoTracker was calculated (n = 3). Data are presented as the mean ± SEM, ^*^P < .05, ^**^P < .01 compared with the control group, ^#^*P* < .05, ^##^*P* < .01 compared with the si-NC group. HRECs = human retinal endothelial cells, ROS = reactive oxygen species.

Oxidative stress is a critical upstream event for activation of the NLRP3 inflammasome.^[33]^ Hence, we investigated whether HOTAIR mediates oxidative stress in DR. Total ROS was increased by high glucose but this was attenuated in HRECs following HOTAIR knockdown (Fig. 4A and B), indicating that HOTAIR may increase ROS generation in DR. Mitochondrial dysfunction, which contributes to the overproduction of mitochondrial ROS (mtROS) and subsequent damage of mitochondrial DNA, is indispensable for NLRP3 inflammasome activation.^[34]^ We assessed the levels of mtROS by MitoSOX staining, a mitochondrial ROS-specific indicator. High glucose increased mtROS but HOTAIR knockdown reduced mtROS generation compared to treatment with si-NC (Fig. 4C and D). Collectively, our results demonstrate that the lncRNA HOTAIR mediates oxidative stress in DR.

### 3.5. HOTAIR inhibits Nrf2 activation

**Figure 5.**
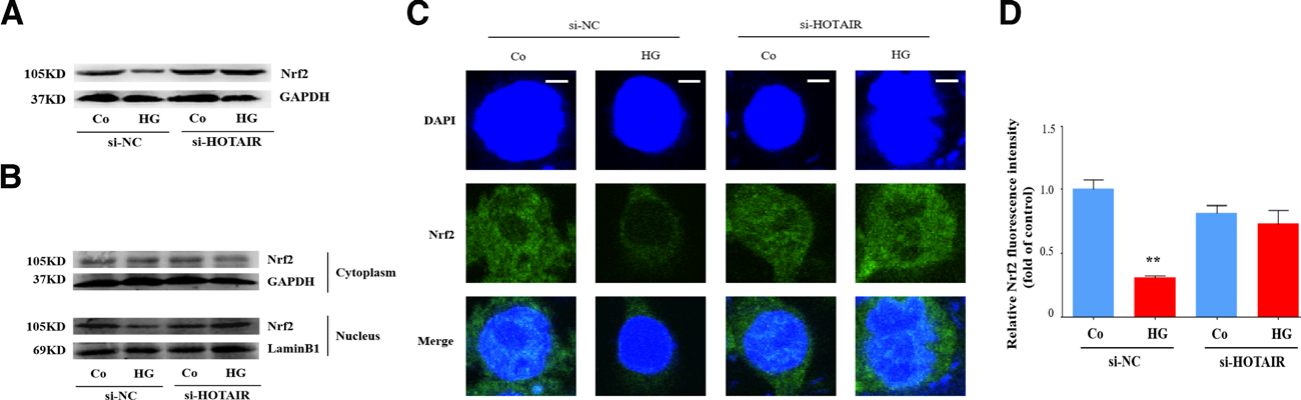
HOTAIR inhibits Nrf2 activation. HRECs were transfected with siRNA-HOTAIR or siRNA-NC, and then incubated with high glucose (HG). (A) Cell lysates were analyzed by immunoblotting to determine the Nrf2 expression. (B) The cytoplasmic and nuclear lysates were analyzed by immunoblotting to determine the Nrf2 expression. (C) The nuclear location of Nrf2 was visualized by immunofluorescence with an anti-Nrf2 antibody. Scale bars, 5 µm. (D) The fluorescence intensity of Nrf2. Data are presented as the mean ± SEM, ^**^P < .01 compared with the control group. HRECs = human retinal endothelial cells, Nrf2 = nuclear factor E2-related factor 2.

To understand the mechanism by which the lncRNA HOTAIR mediates oxidative stress, we measured the effect of HOTAIR on Nrf2 in DR. Nrf2 is an intracellular regulator that stabilizes redox equilibrium and Nrf2 activation protects cells from damage due to oxidative stress.^[35]^ High glucose decreased Nrf2 protein levels but this was ameliorated by HOTAIR knockdown (Fig. 5A). Nrf2 located in the nucleus can drive the transcription of antioxidants and play an antioxidant role.^[35]^ Accordingly, we estimated Nrf2 nuclear movement by detecting the protein level of Nrf2 in cytoplasmic and nuclear lysates by western blotting. High glucose decreased the amount of Nrf2 in the nuclear lysates but HOTAIR knockdown suppressed this decrease (Fig. 5B). Immunofluorescent analysis was used to directly show the location of Nrf2. HOTAIR knockdown enhanced the nuclear translocation of Nrf2 (Fig. 5C and D), which confirms that HOTAIR reduces the nuclear translocation of Nrf2 in DR. According to these results, the lncRNA HOTAIR may inhibit Nrf2 activation in DR.

### 3.6. HOTAIR knockdown enhances the expression of antioxidants modulated by Nrf2

**Figure 6.**
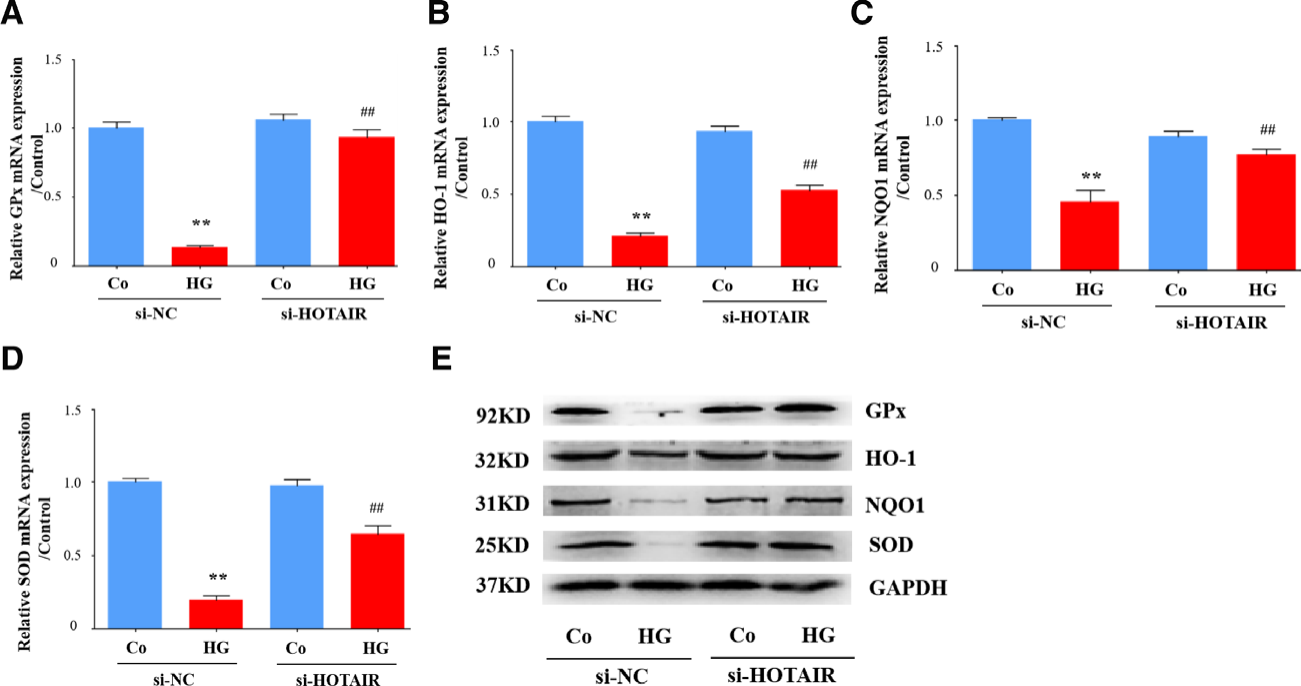
HOTAIR knockdown enhances the expression of antioxidants modulated by Nrf2. HRECs were transfected with siRNA-HOTAIR or siRNA-NC, and then incubated with high glucose (HG). (A–D) The GPx, HO-1, NQO1, and SOD mRNAs were detected by qRT-PCR (n = 3). (E) Cell lysates were analyzed by immunoblotting to determine the GPx, HO-1, NQO1, and SOD protein expression. Data are presented as the mean ± SEM, ^**^P < .01 compared with the control group, ^##^*P* < .01 compared with the si-NC group. HRECs = human retinal endothelial cells, Nrf2 = nuclear factor E2-related factor 2, SOD = superoxide dismutase.

To explore the molecular mechanism in more depth, we focused on antioxidants that are regulated by Nrf2 at the transcriptional level. We measured whether the Nrf2-HO-1 pathway, which eliminates ROS,^[36]^ was modulated by HOTAIR in high glucose-stimulated HRECs. Consistent with the effect of high glucose on Nrf2, we found that high glucose triggered a decline in HO-1; however, knockdown of HOTAIR reversed this decline at both the mRNA and protein level (Fig. 6A and E). GPx, NQO1, and SOD are important antioxidant enzymes that are regulated by Nrf2.^[37]^ High glucose decreased GPx, NQO1, and SOD at both the mRNA and protein level but this decrease was reversed by HOTAIR knockdown (Fig. 6B–E). These results indicate that intracellular antioxidants are impeded by inhibition of Nrf2 via the lncRNA HOTAIR in DR.

### 3.7. HOTAIR promotes the interaction between Nrf2 and Keap1 by binding to Nrf2

**Figure 7.**
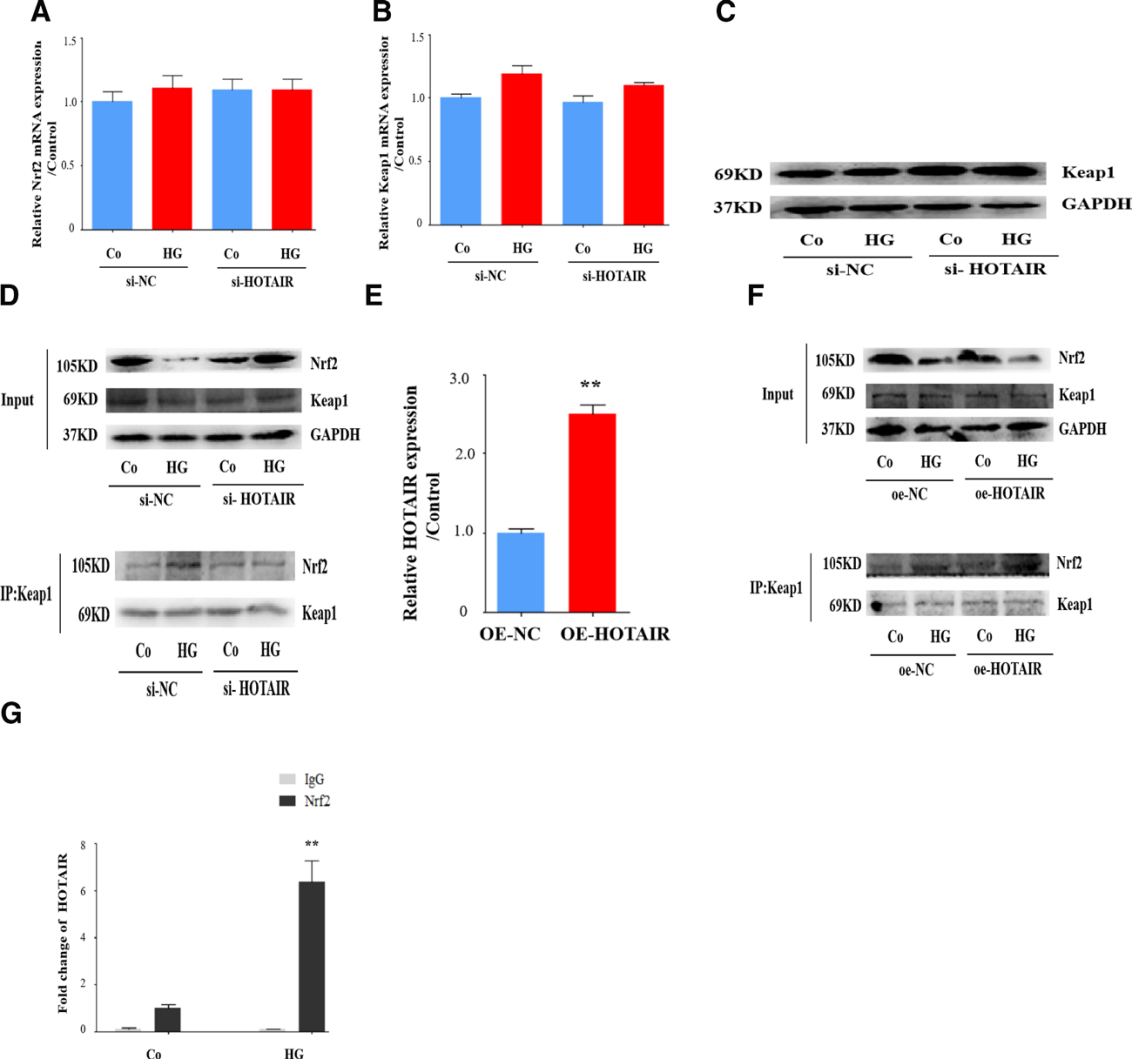
HOTAIR promotes the interaction between Nrf2 and Keap1 by binding to Nrf2. HRECs were transfected with siRNA-HOTAIR, oe-HOTAIR, or siRNA-NC, and then incubated with high glucose (HG). (A–B) The Nrf2 and Keap1 mRNAs were detected by qRT-PCR (n = 3). (C) Cell lysates were analyzed by immunoblotting to determine the Keap1 protein expression. (D–E) The interaction between Nrf2 and Keap1 was analyzed by immunoprecipitation using an antibody against Keap1 and then measured by immunoblotting. (F) HRECs were incubated with HG. The chromatin was immunoprecipitated with control IgG or an anti-Nrf2 antibody, and then cDNA was synthesized. The lncRNA HOTAIR level was quantified by qRT-PCR (n = 3). Data are presented as the mean ± SEM, ^**^P < .01 compared with the control group. HRECs = human retinal endothelial cells, Nrf2 = nuclear factor E2-related factor 2.

Next, we examined how HOTAIR regulates Nrf2 inactivation in high glucose-stimulated HRECs. In contrast to the Nrf2 protein level, high glucose treatment did not reduce the Nrf2 mRNA level and the knockdown of HOTAIR did not affect the Nrf2 mRNA (Fig. 7A), indicating that the inhibition of Nrf2 activation by HOTAIR not result from a decrease in *Nrf2* expression.

We next analyzed the expression of Kelch-like ECH-associated protein 1 (Keap1), a binding protein of Nrf2. Activation of Nrf2 depends on the dissociation of the binding to Keap1.^[38]^ High glucose had little effect on Keap1 at the mRNA and protein levels (Fig. 7B and C). As the expression of Nrf2 and Keap1 expression were barely changed by high glucose treatment, we employed HOTAIR knockdown and overexpression followed by co-immunoprecipitation using a specific antibody to Keap1 to test whether HOTAIR affected the interaction between Nrf2 and Keap1. Co-immunoprecipitation assays revealed that high glucose strengthened the interaction between Nrf2 and Keap1 in HRECs and that HOTAIR knockdown partly attenuated this interaction (Fig. 7D). In contrast, the interaction between Nrf2 and Keap1 was enhanced upon HOTAIR overexpression (Fig. 7E and F). The binding between Nrf2 and lncRNA is a reliable way to exhibit the biological function of Nrf2.^[38]^ We tested the binding between Nrf2 and HOTAIR using a ChIP assay. Significant enrichment of HOTAIR was detected in chromatin material precipitated with an anti-Nrf2 antibody in HRECs stimulated with high glucose (Fig. 7G). These results suggest that the lncRNA HOTAIR promotes the interaction between Nrf2 and Keap1 by binding to Nrf2.

### 3.8. The HOTAIR-mediated promotion of ROS and NLRP3 activation is dependent on the inhibition of Nrf2

**Figure 8.**
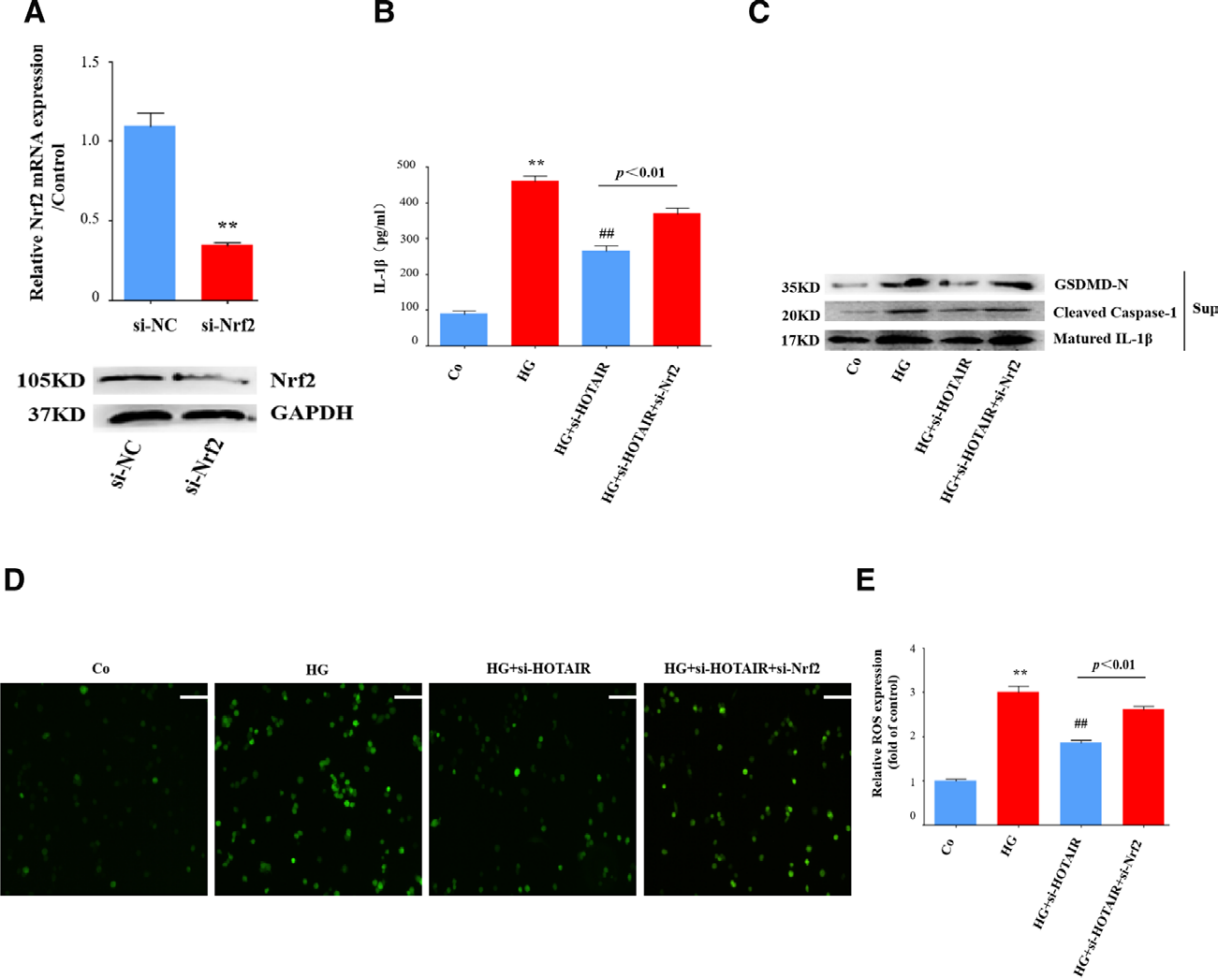
HOTAIR promotes ROS generation and NLRP3 activation via inhibition of Nrf2. HRECs were transfected with siRNA-HOTAIR or siRNA-Nrf2 (A) and then incubated with high glucose (HG). (B) Supernatants were analyzed by ELISA to measure IL-1β (n = 3). (C) Supernatants were analyzed by immunoblotting to determine the release of cleaved caspase-1, mature IL-1β, and GSDMD-N. (D and F) Cells were stained with DCFH (10 μM) and then analyzed by florescent microscopy and using a microplate reader (n = 3). (E) HRECs were transfected with OE-NC or OE-HOTAIR. (G) The chromatin were immunoprecipitated with control (IgG) or Nrf2-specific antibody followed by the reversal of the cross-linking and DNA isolation. DNA was used in quantitative PCR with primers specific for HOTAIR (n = 3). Scale bars, 50 µm. ^*^P < .05, ^**^P < .01 compared with the control group, ^#^*P* < .05, ^##^*P* < .01 compared with the HG group. Nrf2 = nuclear factor E2-related factor 2, ROS = reactive oxygen species.

As the above results suggest that HOTAIR knockdown enhances Nrf2 activation, we explored whether decreased Nrf2 is required for the action of HOTAIR. We employed a strategy that knock down Nrf2 with a si-RNA (Fig. 8A). Knockdown of Nrf2 reversed the HOTAIR-knockdown-mediated decrease of IL-1β levels and NLRP3 inflammasome activation (Fig. 8B and C). Similarly, Nrf2 knockdown also neutralized the HOTAIR-knockdown-mediated inhibition of ROS generation (Fig. 8D and E). These data suggest that the decrease in Nrf2 caused by the lncRNA HOTAIR is indispensable for mediating the oxidative stress and NLRP3 inflammasome activation induced by high glucose in HRECs.

## 4. Discussion

The current study suggests that the lncRNA HOTAIR is involved in DR. HOTAIR was overexpressed in the vitreous of patients with DR and in HRECs stimulated with high glucose. Knockdown of HOTAIR depressed oxidative stress and subsequent NLRP3 inflammasome activation in HRECs treated with high glucose. Furthermore, HOTAIR reduced intracellular antioxidants by inhibiting Nrf2. Mechanistically, HOTAIR enhanced the interaction between Nrf2 and Keap1 by binding to Nrf2. Therefore, our findings identify a previously undescribed mechanism and a potential therapeutic target for DR.

HOTAIR is a critical lncRNA in inflammatory diseases, including gout,^[39]^ ischemia-reperfusion injury,^[40]^ and diabetes,^[22]^ and is associated with oxidative stress, cytokines release, and apoptosis. HOTAIR is a potential therapeutic target against inflammatory diseases.^[41,42]^ HOTAIR knockdown attenuates nucleus pulposus cell death thus preventing intervertebral disc degeneration.^[41]^ However, a previous study suggested that HOTAIR is decreased and that enhancing HOTAIR has a protective effect in rheumatoid arthritis.^[42]^ These reports showed that the biological function of HOTAIR is diverse in different diseases. Biswas et al reported that HOTAIR is upregulated in the serum and vitreous of patients with DR.^[43]^ Consistent with it, we also found higher levels of HOTAIR in the vitreous of patients with DR compared to controls, and in vitro data confirmed this result. Furthermore, HOTAIR correlated with oxidative stress and inflammation in DR. Collectively, these data conclude that the lncRNA HOTAIR may promote DR development.

The NLRP3 inflammasome is an essential regulator of the immune and inflammatory responses.^[5–8]^ Upon activation, the activated NLRP3 inflammasome serves as a platform for the maturation and release of proinflammatory mediators, subsequently leading to a series of physiological effects.^[6,7]^ In the pathological process of DR, NLRP3 inflammasome activation can trigger the generation of inflammatory factors and subsequent dysfunction and proliferation of retinal endothelial cells.^[8,31,44]^ We found that HOTAIR knockdown can inhibit NLRP3 inflammasome activation induced by high glucose in this study. HOTAIR promotes NLRP3 inflammasome expression in THP-1 cells induced by monosodium urate.^[39]^ The expression of NLRP3, pro-IL-1β, and pro-caspase-1 in an NF-κB-dependent manner triggers NLRP3 inflammasome activation.^[5,45,46]^ As a harmful stimulus, high glucose can provoke NLRP3 inflammasome activation and subsequent physiological effects in this way.^[47]^ We found that HOTAIR knockdown could depress the expression of NLRP3, pro-caspase-1, and pro-IL-1β at both the mRNA and protein levels. According to these findings, we propose that the lncRNA HOTAIR facilitates NLRP3 inflammasome activation in DR.

Oxidative stress is a critical upstream event for NLRP3 inflammasome activation.^[48]^ Abnormal ROS production is a signaling intermediate that can drive NLRP3 inflammasome expression via the NF-κB pathway.^[49]^ Furthermore, ROS induced by high glucose are involved in the inflammatory process in DR and it may be feasible to prevent DR by inhibiting the generation of ROS.^[10,50]^ The mitochondrial ROS is the most direct indicator for mitochondrial damage, and the augment of mitochondrial ROS can trigger the activation of NLRP3 inflammasome.^[51]^ Here, we found that HOTAIR mediates ROS generation induced by high glucose in HRECs, and triggers mitochondrial damage. Together, these data indicate that HOTAIR promotes ROS-dependent NLRP3 inflammasome activation in DR.

Nrf2 protects cells as part of the inflammatory response under oxidative stress.^[14,15]^ Activated Nrf2 accelerates antioxidants expression and initiates related antioxidant systems.^[12,13]^ Studies have demonstrated that activating Nrf2 is the chief measure for some cell protectors to develop antioxidant and subsequent anti-inflammatory roles.^[12,13]^ Correspondingly, the inactivation of Nrf2 may lead to oxidative damage.^[52]^ Depression of Nrf2 delays diabetic wound healing owing to dysfunction of the antioxidant system.^[26]^ A recent study reported that high glucose elevates cytosolic accumulation of Nrf2 and prevents Nrf2 activation.^[24]^ The Nrf2 activator can suppress oxidative stress responses induced by high glucose in human retinal epithelial cells.^[53]^ Here, we show that HOTAIR knockdown reversed the high glucose-induced inactivation of Nrf2 and the decrease in expression of antioxidants dependent on Nrf2. The activation of the Nrf2/HO-1 pathway can prevent high glucose-mediated damage in retinal pigment epithelial cells.^[54]^ Moreover, the present study found that the inactivation of Nrf2 is required for HOTAIR-mediated oxidative stress and NLRP3 inflammasome activation. LncRNA functions via interacting with target proteins.^[55]^ HOTAIR binds to histone-modifying enzymes to regulate DR-related molecules that are dependent on glycolytic metabolism.^[23]^ According to these findings, we propose that the lncRNA HOTAIR mediates ROS generation by inactivating Nrf2 in DR. However, the activation of NADPH oxidase also promotes ROS generation and induces oxidative injury. So, the ROS from NADPH oxidase whether promotes the lncRNA HOTAIR/ROS/NLRP3/Nrf2 pathway should be further studied in DR in the future.

In summary, the present study demonstrates that HOTAIR is involved in DR. Importantly, we showed that HOTAIR inhibits Nrf2 and promotes ROS generation, thus facilitating NLRP3 inflammasome activation in DR. This previously undescribed regulatory mechanism may provide a novel target for the treatment of DR. However, some limitations are existed in the present study. We did not explored the relationship between LncRNA HOTAIR and IL-1β, IL-18, SOD and MDA in serum of patients with DR. The depolarization of mitochondrial membrane potential has not been studied. In addition, we did not employed animal model to validate the mechanism in vivo. In the future, we will measure the levels of IL-1β, IL-18, SOD and MDA, and employ animal experiment to deeply explore the function of LncRNA HOTAIR on DR.

## Acknowledgments

We thank LetPub (www.letpub.com) for its linguistic assistance during the preparation of this manuscript.

## Author contributions

**Data curation:** Hongyu Li.

**Investigation:** Hui You, Wenjun Gou.

**Methodology:** Hui You, Hongyu Li, Wenjun Gou.

**Project administration:** Wenjun Gou.

**Writing – original draft:** Hui You.

**Writing – review & editing:** Wenjun Gou
